# Assembly and analysis of the complete mitochondrial genome of the Chinese wild dwarf almond (*Prunus tenella*)

**DOI:** 10.3389/fgene.2023.1329060

**Published:** 2024-01-11

**Authors:** Xingyue Liu, Dongdong Zhang, Zhenfan Yu, Bin Zeng

**Affiliations:** College of Horticulture, Xinjiang Agricultural University, Urumqi, China

**Keywords:** *Prunus tenella*, mitochondrial genome, phylogenetic, RSCU, *Prunus*

## Abstract

**Background:** The wild dwarf almond (*Prunus tenella*) is one of the national key grade II-protected wild plants in China. It is a relic deciduous forest species from the middle Eocene of the ancient Mediterranean Sea and is also known as a “living fossil of plants.” It is distributed in Southeast Europe, West Asia, Central Asia, Siberia, and Xinjiang (Tacheng) and other areas of China. The plant grows on arid slopes, steppes, depressions, and valleys at an altitude of 1,200 m. The seeds of wild dwarf almonds are frost resistant and contain oil and bitter lentil glycosides, which possess medicinal value. Additionally, the seeds of wild dwarf almonds can be used as the original material for breeding new varieties of almonds and obtain ornamental flowers and trees.

**Results:** The complete mitochondrial genome of *P. tenella* was sequenced and assembled using two sequencing platforms, namely, Illumina Novaseq6000 and Oxford Nanopore PromethION. The assembled genome was 452,158-bp long with a typical loop structure. The total number of A, T, C, and G bases in the genome was 122,066 (26.99%), 124,114 (27.45%), 103,285 (22.84%), and 102,693 (22.71%), respectively, with a GC content of 45.55%. A total of 63 unique genes, including 36 protein-coding genes, 24 tRNA genes, and 3 rRNA genes, were identified in the genome. Furthermore, codon usage, sequence duplication, RNA editing, and mitochondrial and chloroplast DNA fragment transfer events in the genome were analyzed. A phylogenetic tree was also constructed using 30 protein-coding genes that are common to the mitochondrial genomes of 24 species, which indicated that the genome of wild lentils is highly conserved with those of apples and pears belonging to Rosaceae.

**Conclusion:** Assembly and annotation of the *P. tenella* mitochondrial genome provided comprehensive information about the mitochondrial genome of wild dwarf almonds, This study provides information on the mitochondrial genome of *Prunus* species and serves as a reference for further evolutionary studies on wild dwarf almonds.

## Introduction

*Prunus tenella*, a relic of the Quaternary Ice Age, originated in Central Asia during the Eocene epoch of the tertiary period (Sorkheh et al., 2009), and it is found only in locally warmer and wetter areas 800–1,300 m above sea level. The species exhibits a discontinuous island-like distribution, with the Mediterranean coast, the Middle East, and China as the main distribution centers. Because it evolved in the ice age, *P. tenella* is almost extinct at present, with only a few remnants in the world, making it a precious species. In China, *P. tenella* is mainly distributed in Xinjiang, a region of the Balluk Mountains on the border of Yumin County. *Prunus* species are highly adaptable, with strong cold tolerance, and can be safely overwintered at −30 °C (Zeng et al., 2019). Moreover, they can be used as a cold-resistant dwarf rootstock for fruit trees, such as peaches, *Prunus,* and wild dwarf almonds. Furthermore, *P. tenella* seed kernels contain 51% oil, of which 80% is oleic acid that is required by humans. The oil residue contains 28% protein, which can be consumed or used as high-grade feed (Wang et al., 2019).

The genus *Prunus* comprises various species such as *P*. *persica*, *P*. *salicina*, *P*. *armeniaca*, and *P*. *dulcis*. Research on the evolution and classification of *Prunus* species in the world has yielded valuable results. For instance, clustering based on chloroplast ndhF and ITS sequences showed that *P. salicina* is closely related to *P. persica*, and both are distant from *P*. *pseudocerasus* (Jun et al., 2008). Analysis of *Prunus* using the ITS sequence of ribosomal DNA indicated that *P*. *mume* is more closely related to *P. salicina* than *P. armeniaca* (Lee and Wen, 2001a). Based on chloroplast DNA sequence clustering, a close relation of *P*. *domestica* and *P*. *insititia* with *P*. *cerasifera* was reported (Reales et al., 2010). According to the modern molecular classification system, plants of the genus *Amygdalae* should belong to the genus *Rosaceae*, the subfamily *Amygdaloideae*, the tribe *Amygdaleae*, and the genus *Prunus* L. sensu lato (Potter et al., 2007). In China, almonds belong to two major groups, cultivated and wild dwarf. Among them, wild lentils are of five types, namely, *P. tenella*, *P*. *mongolica*, *P*. *tangutica*, *P*. *trilob*, and *P*. *pedunculata*. The results of chloroplast genome clustering showed that the three species *P. tenella*, *P. pedunculata*, and *P. triloba* were highly conserved as they clustered together in the same group, whereas *P. dulcis* and *P. persica* clustered in another group (Lin et al., 2022). In terms of geographic distribution, *P. tenella*, *P. pedunculata*, and *P. triloba* are distributed at higher latitudes, while *P. mongolica* and *P. tangutica* are distributed at relatively low latitudes (Wang et al., 2021). Furthermore, exploiting mitochondrial genome sequence conservation is a common and effective tool for phylogenetic analyses of species.

The mitochondrion, also known as the powerhouse of the cell, is a two-layered organelle found in most cells and is the major site of aerobic respiration (Birky, 1995; Ye et al., 2017). Most eukaryotic cells contain mitochondria, which differ in size, number, and characteristics across living organisms (Greiner and Bock, 2013). The mitochondrial genome mainly contains one chromosome with abundant polymorphism and has its own genetic material and genetic system. However, its genome size is limited, and it is a semi-autonomous organelle (Jacoby et al., 2012). In addition to supplying energy to cells, mitochondria are involved in cellular processes such as cell differentiation, cellular information transfer, and apoptosis and can regulate cell growth and cell cycle (Møller et al., 2021). In most plants, nuclear genetic information is inherited from both parents, whereas the mitochondrial and chloroplast DNA are inherited maternally. The mitochondrial genomic sequences of plants can vary significantly in length, mostly between 100 Kb and 12 Mb, and possess 60–70 conserved genes involved in oxidative phosphorylation and protein translation and many sequence regions of unknown function (Sloan et al., 2012; Bi et al., 2020a).

The mitochondrial genomes of many species of *Prunus* in the family Rosaceae have been published. The study of mitochondrial genomes of *Prunus* species can provide valuable molecular data and aid in the study of the genetic evolution of *Prunus* species. To date, *P. tenella* mitochondrial genome-related studies are lacking. Therefore, we assembled the complete mitochondrial genome of *P. tenella* and analyzed relative synonymous codon usage (RSCU), repetitive sequences, selection pressure, RNA editing sites, and phylogenetic relationships. Furthermore, we investigated the nucleic acid diversity of the *P. tenella* mitochondrial genome, comparative mitochondrial structures, and gene transfer between the chloroplast and mitochondrial genomes. To the best of our knowledge, this study is the first to provide the complete mitochondrial genome of *P. tenella* by combining second- and third-generation sequencing approaches. A series of mitochondrial features were also analyzed to provide molecular biological data for taxonomic studies of *Prunus* species.

## Materials and methods

### Material preparation and genome sequencing

*P. tenella* “Yumin” branches were collected from the Sanping Teaching Practice Base of Xinjiang Agricultural University on 12 April 2022. The collected branches were brought to the laboratory, and their leaves were used to extract high-quality genomic DNA by using a modified CTAB method. The quality and quantity of the extracted DNA were evaluated using a NanoDrop 2000 spectrophotometer (NanoDrop Technologies, Wilmington, DE, United States), Qubit dsDNA HS Assay Kit on a Qubit 3.0 Fluorometer (Life Technologies, Carlsbad, CA, United States), and electrophoresis on a 0.8% agarose gel, respectively. The SMRTbell library was constructed using the SMRTbell Express Template Prep Kit 2.0 (Pacific Biosciences) (Zhang et al., 2022). Briefly, 15 μg of the genomic DNA was used for the first enzymatic reaction to remove single-stranded overhangs, followed by the treatment with repair enzymes to repair potentially damaged sites on the DNA backbone. Afterward, the ends of the double-stranded fragments were polished and subsequently tailed with an A-overhang. Then, ligation with T-overhang SMRTbell adapters was performed at 20°C for 60 min T, and the SMRTbell library was purified using 1X AMPure PB beads. The size distribution and concentration of the library were assessed using a FEMTO Pulse automated pulsed-field capillary electrophoresis instrument (Agilent Technologies, Wilmington, DE) and a Qubit 3.0 Fluorometer (Life Technologies, Carlsbad, CA, United States), respectively (Chang et al., 2021). Following library characterization, 3 μg of the genomic DNA was subjected to a size selection step using the BluePippin system (Sage Science, Beverly, MA) to remove SMRTbells with size ≤ 15 kb. Then, the library was purified using 1X AMPure PB beads. Library size and quantity were assessed using the FEMTO Pulse and the Qubit dsDNA HS reagents assay kit, respectively. Sequencing primer and Sequel II DNA Polymerase were annealed and bound, respectively, to the final SMRTbell library. Subsequently, sequencing was performed on a PacBio Sequel II platform at a concentration of 120 p.m., with a running time of 30 h (HolmFranceMa et al., 2019). SMRT sequencing was performed using a single 8 M SMRT Cell on the Sequel II System with Sequel II Sequencing Kit and 1800-min movies produced by Frasergen Bioinformatics Co., Ltd. (Wuhan, China) (Supplementary Table S1).

### Genome assembly and annotation

Plant mitochondrial genes (coding sequence [CDS] and rRNA) are highly conserved. Therefore, the original long-read sequencing data were compared with the reference gene sequence (plant mitochondrial core gene) by using Minimap2 (version: 2.1) (Li, 2018); the sequence with a similar fragment longer than 50 bp was selected as the candidate sequence, and candidate sequences with more aligned genes (one sequence containing multiple core genes) and higher alignment quality (covering more complete core genes) were selected as the seed sequence. The original long-read sequencing data were then compared with the seed sequence. The sequences with a minimum overlap of 1 kb and at least 70% similarity were added to the seed sequence. The original data were iteratively aligned with the seed sequence to obtain all long-read sequencing data of the mitochondrial genome.

Using the assembly software Canu, the long-read sequencing data were corrected. Then, Bowtie2 (version: 2.3.5.1) was used to align the short-read sequencing data to the corrected sequence. The default parameter Unicycler (version: 0.4.8) was used to compare the abovementioned short-read sequencing data with the corrected long-read sequencing data for concatenation (Wick et al., 2017). Finally, the ring *P*. *tenella* mitochondrial genome was obtained.

The annotation of mitochondrial genome structure was performed in the following steps: (1) For the encoding protein and rRNA, the basic local alignment search tool (BLAST) was used to align the published plant mitochondrial sequences as “refs.” Further manual adjustments were made for related species. (2) Transfer RNA (tRNA) was annotated using tRNAs-canSE (http://lowelab.ucsc.edu/tRNAscan-SE/) (Chan and Lowe, 2019). (3) Open Reading Frame Finder (https://www.ncbi.nlm.nih.gov/gorf/gorf.html) was used to annotate open reading frames, with the shortest length set to 102 bp. Redundant sequences and those overlapping with known genes were excluded. Sequence alignments greater than 300 bp were annotated to the NR library. The abovementioned results were verified and manually corrected to obtain highly accurate annotation results. Lastly, the mitochondrial genome was mapped using OGDRAW (https://chlorobox.mpimp-golm.mpg.de/OGDraw.html).

### Characterization of repeat sequences and protein-coding genes

Three types of repeats (simple sequence [SSRs], tandem, and dispersed) were identified in the *P*. *tenella* mitochondrial genome. The MIcroSAtellite (MISA) identification tool Perl script (version: 1.0, parameter: 1-10 2-5 3-4 4-3 5-3 6-3) was used to detect SSRs (Thiel et al., 2003). Tandem repeats (consisting of repeat units longer than 6 bp) were detected using Tandem Repeats Finder (version: 4.09) software (http://tandem.bu.edu/trf/trf.submit.options.html) (trf409. linux64, parameter: 2 7 7 80 10 50 2000 -f -d -m), with default settings (Benson, 1999). Dispersed repeats were identified using blastn (version: 2.10.1), with the following parameters: word size, 7; e-value, 1e-5; removal of redundant sequences and tandem duplication. These repeats were visualized using Circos v. 0.69–5 (Krzywinski et al., 2009). In addition, repetitive sequences with a length of ≥50 bp and a similarity of 100% were identified using vmatch (version: 2.3.0) software (Kimura, 1980). To examine the extent of nucleic acid sequence variation in the *P. tenella* mitochondrial genome, homologous gene sequences from different species were globally compared using MAFFT software (version: 7.427, --auto mode). Subsequently, pi (nucleic acid diversity) values were calculated for each gene by using DnaSP5 (Librado and Rozas, 2009).

### DNA transfer from chloroplasts to mitochondria

The *P. tenella* chloroplast genome (GenBank: MH727487.1) was obtained from the NCBI Organelle Genome Resources Database. BLAST software was used to identify homologous genes and tRNA genes transferred from the chloroplasts to mitochondria, with the following screening criteria: matching rate, 70%; e-value, 1e-5; and minimum length, 30 bp. The homologous fragments between the chloroplast and mitochondrial genomes were visualized using Circos (version: 0.69–5) (Krzywinski et al., 2009).

### Phylogenetic analysis

For the phylogenetic clustering analysis, 24 plant species from the families Cruciferae, Leguminosae, Solanaceae, Gramineae, Rosaceae, Asteraceae, Umbelliferae, and Ginkgoaceae were selected, and phylogenetic trees were constructed using *P. tenella* mitochondrial genomes (Supplementary Table S2). A maximum likelihood evolutionary tree was constructed using the CDS of the mitochondrial genomes of all species. The sequences between species were aligned using the MAFFT software (version: 7.427, --auto mode). Prior to alignment, the sequences were concatenated, and then trimmed using trimAl (version: 1.4. rev15) with the parameter -gt 0.7. Jmodeltest (version: 2.1.10) software was used to predict the model type, determining it as the GTR type. Finally, the maximum likelihood evolutionary tree was constructed using RAxML (version: 8.2.10) (https://cme.h-its.org/exelixis/software.html) with the GTRGAMMA model and a Bootstrap value = 1,000 (Supplementary Table S3) (Capella-Gutiérrez et al., 2009; Stamatakis, 2014). We used the same method to construct the phylogenetic tree of eight *Prunus* species, namely, *P. tenella*, *P*. *sibirica* (ON478172.1), *P*. *mume* (ON478169.1), *P*. *armeniaca* (ON478164.1), *P*. *salicina* (OK563724.1), *P*. *avium* (ON478178. 1), *P*. *mira* (ON478168.1), and *P*. *dulcis* (CM038036.2).

### Selection of evolutionary pressure

The mitochondrial genomes of seven *Prunus* species were selected to extract 20 protein-coding genes (*atp1, atp4, atp6, ccmFc, ccmFn, cob, cox2, cox3, matR, mttB, nad1, nad2, nad4, nad5, nad6, nad7, rpl5, rps1, rps3*, and *rps4*) in pairwise combinations. Homologous gene pairs were aligned using the MAFFT (version: 7.310) (https://mafft.cbrc.jp/alignment/software/) software. After alignment, KaKs_Calculator (version: 2.0) (https://sourceforge.net/projects/kakscalculator2/) was employed to calculate *Ka* and *Ks* values for each gene pair by using the MLWL calculation method (Wang et al., 2010).

## Results

### Characterization of the mitochondrial genome of *P. tenella*

**FIGURE 1:**
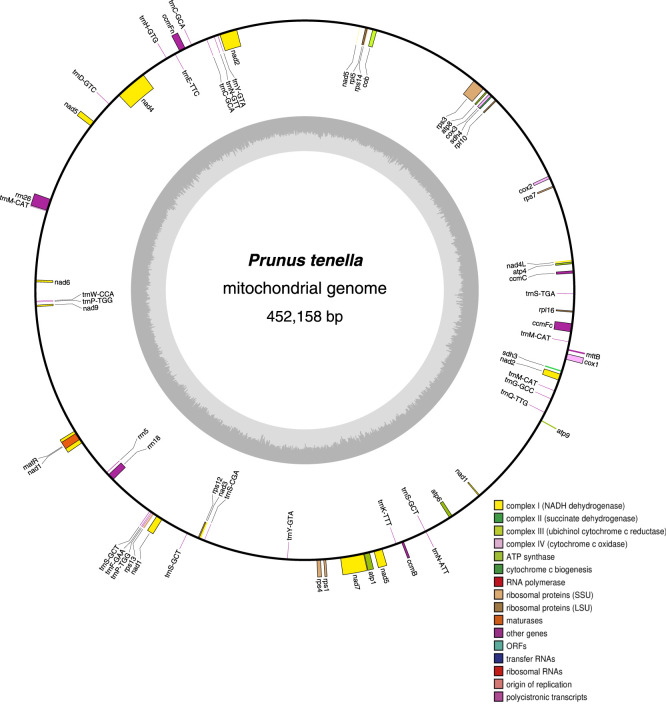
Loop structure of the *P. tenella* mitochondrial genome. The inner black circle indicates the clockwise distribution of genes, whereas the outer black circle indicates the counterclockwise distribution. Different colors represent genes with distinct functions, and the dark gray diagram in the inner circle represents the GC content.

The mitochondrial genome of *P. tenella* was found to have a typical loop structure, with a total sequence length of 452,158 bp (GenBank accession number: OQ559502.1). Figure 1 illustrates the gene distribution and functional classification of all identified genes in the *P. tenella* mitochondrial genome. The base composition of the genome was as follows: 122,066 (26.99%) A bases, 124,114 (27.45%) T bases, 103,285 (22.84%) C bases, and 102,693 (22.71%) G bases, with the GC content being 45.55%. A total of 63 unique genes, including 36 protein-coding, 24 tRNA, and 3 rRNA genes, were identified in the genome. Notably, three tRNA genes (*trnC-GCA*, *trnP-TGG*, and *trnY-GTA*) had two copies each, and two tRNA genes (*trnM-CAT* and *trnS-GCT*) had three copies (Supplementary Table S4).

### Analysis of codon usage in protein-coding genes

**FIGURE 2:**
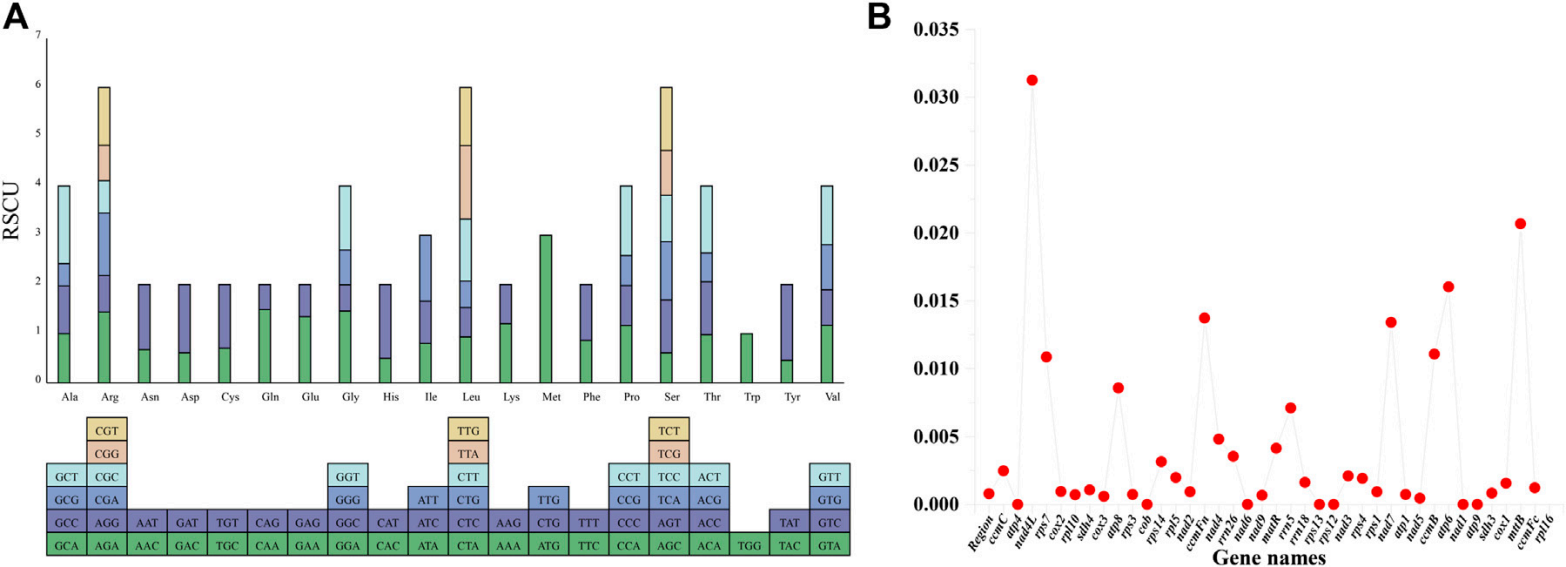
*P. tenella* mitochondrial genome RSCU and pi values. **(A)** Distribution of RSCU values for protein-coding genes. Each amino acid is represented by a square containing all the codons encoding it, and the height of the bar above indicates the sum of RSCU values for all codons. **(B)** Line graph displaying the pi values for 39 genes. Gene names are represented on the horizontal axis, and pi values are indicated on the vertical axis.

Of the 36 protein-coding genes identified in the genome, 33 were found to have ATG as the start codon, 2 (*nad1* and *rps4*) had ACG, and 1 (*rpl16*) had GTG as the start codon. The stop codons were TAA, TAG, and TGA in 20, 7, and 9 of the 36 protein-encoding genes, respectively, indicating the absence of C-to-U RNA editing events in the stop codons (Supplementary Table S4). The RSCU of the protein-coding genes in the *P. tenella* mitochondrial genome was also analyzed (Supplementary Table S5). The results revealed that the 36 protein-coding genes, totaling 30,651 bp, encode 10,217 codons, including stop codons (Supplementary Table S4). Among these, 32 are high-frequency codons (RSCU > 1), and except four codons, UUG (Leu), AUG (Met), ACC (Met), and UGG (Trp), all codons had A or U as the third base. Additionally, two Met codons (CUG and UUG) were assigned a value of 0 (Figure 2A). Nucleic acid diversity was calculated for 39 genes (Figure 2B). The pi value of *rsp7* was found to be 0.03126, whereas those of *atp1, atp6, cox2, ccmFc, nad1*, and *nad4* were greater than 0.01.

### Repeat sequence analyses of the mitochondrial genome of *P. tenella*

**FIGURE 3:**
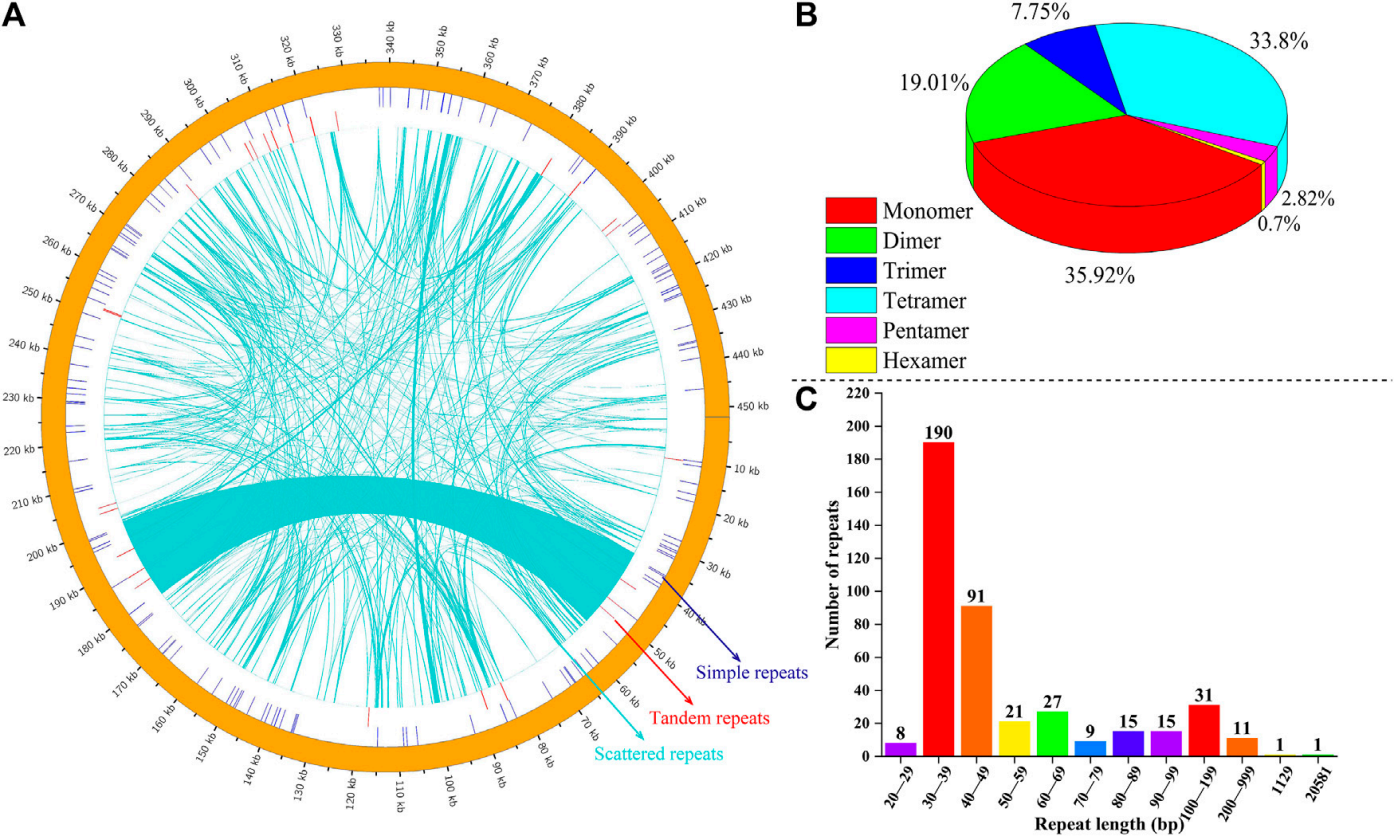
Mitochondrial genomic repeat sequences of *P. tenella*. **(A)** Distribution of repetitive sequences on the genome. The outermost circles represent simple sequence repeats, followed by tandem repeats, and the innermost contigs represent scattered repeats. **(B)** Statistical map of the five types of SSRs in the *P. tenella* mitochondrial genome. Different colors represent different types of SSRs. **(C)** Statistical histogram of the *P. tenella* mitochondrial genome with repetitive sequences of different lengths. The horizontal axis represents the length range, and the vertical axis represents the number of repetitive sequences.

We analyzed SSRs, tandem repeats, and dispersed repeats in the mitochondrial genome of *P. tenella*; the distribution of these repeat sequences are depicted in Figure 3A. A total of 142 SSRs were identified, including 51 (35.92%) monomers, 27 (19.01%) dimers, 11 (7.75%) trimers, 48 (33.80%) tetramers, 4 (2.82%) pentamers, and 1 (0.70%) hexamer (Figure 3B). Further analyses of the SSR replicate units revealed that the A/T content in the monomers and tetramers was 39 (76.47%) and 119 (52.19%), respectively, and the G/C content was 12 (23.53%) and 73 (32.02%), respectively. Most SSRs were distributed in the spacer region sequences, and only three SSRs, *nad1* (monomer), *rps1* (trimer), and *rps3* (tetramer), were found to be in the protein-coding regions (Supplementary Table S6). We also identified 26 pairs of tandem repeats with sequence lengths ranging from 14 to 56 bp, all of which were situated in the spacer regions. Among them, *cox2*/*rpl10*, *nad5*/*rrn26*, *trnS-CGA*/*trnY-GTA*, and *nad1*/*atp9* contained three tandem repeats in their respective spacer regions, while *rps3*/*cob*, *trnM-CAT*/*nad6*, *nad1*/*nad9*, and *nad1*/*trnS-GCT* had two tandem repeats. Moreover, *nad4L*/*rps7*, *nad5*/*nad2*, *rrn18*/*trnS-GCT*, *trnS-GCT*/*rps12*, *nad3*/*trnS-CGA*, and *atp6*/*nad1* had one tandem repeat each (Supplementary Table S7). Furthermore, we identified 421 dispersed repeats in the *P. tenella* mitochondrial genome, with a total length of 47,009 bp, accounting for 10.39% of the overall genome (Figure 3C; Supplementary Table S8). Sequences less than 1 kb in length are considered short repeat sequences and those greater than 1 kb are large repeat sequences. The length of 190 (45.13%) dispersed repeats ranged from 30 to 39 bp, that of 91 (21.62%) dispersed repeats ranged from 40 to 49 bp, that of 31 (7.63%) dispersed repeats ranged from 100 to 199 bp, and that of 11 (2.61%) dispersed repeats ranged from 200 to 999 bp. Notably, two dispersed repeats exceeded 1 kb in length, one measuring 1,129 bp and the other 20,581 bp. In addition, we identified 24 forward and 22 palindromic repeat sequences in the *P. tenella* mitochondrial genome (Supplementary Figures S1, 2; Supplementary Table S8).

### Editing sites of the *P. tenella* mitochondrial genomic RNA

**FIGURE 4:**
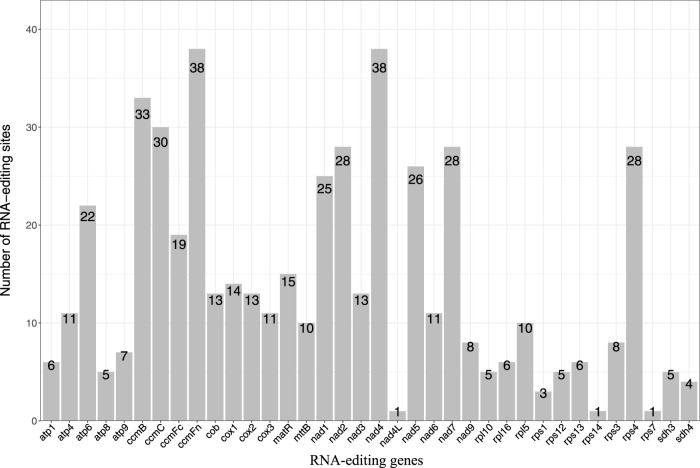
Statistical plot of the number of RNA editing sites in protein-coding genes of the *P. tenella* mitochondrial genome. The horizontal axis represents the genes, and the vertical axis represents the number of editing sites.

We identified a total of 507 RNA editing sites in the 36 protein-coding genes of the *P. tenella* mitochondrial genome, all exhibiting a C-to-U(T) RNA editing pattern (Figure 4; Supplementary Table S9). The analysis revealed that *ccmFn* and *nad4* had the highest number of RNA editing sites (37 sites each), whereas *nad4L, rps14*, and *rps7* had only one RNA editing site each. Among the genes, 15 had fewer than 10 RNA editing sites, 11 had 10–19 sites, 6 had 20–29 sites, and 4 had 30–39 sites. Further examination of the RNA editing sites indicated that 173 (34.12%) sites occurred at the first base of the codon, 350 (69.03%) sites occurred at the second base, and 16 (3.16%) occurred at both the first and second bases. No RNA editing sites were found at the third base. The amino acid changes at the editing sites were: (1) serine (185) to phenylalanine (66) and leucine (119), (2) proline (166) to phenylalanine (16), leucine (111), and serine (39) (Supplementary Table S9). Additionally, an analysis of the codon amino acids affected by RNA editing (Supplementary Table S10) showed that 64 (12.62%) editing sites resulted in the conversion of hydrophilic amino acids to hydrophilic ones; hydrophilic to hydrophobic conversion occurred in 247 (48.72%) sites; hydrophobic to hydrophilic conversion occurred in 39 (7.69%) sites; and hydrophobic to hydrophobic conversion occurred in 155 (30.57%) sites. Notably, a conversion of glutamine to a stop codon was observed in *atp6*, and a conversion of arginine to a stop codon was observed in *ccmFc*.

### Identification of homologous sequences in the chloroplast and mitochondria

**FIGURE 5:**
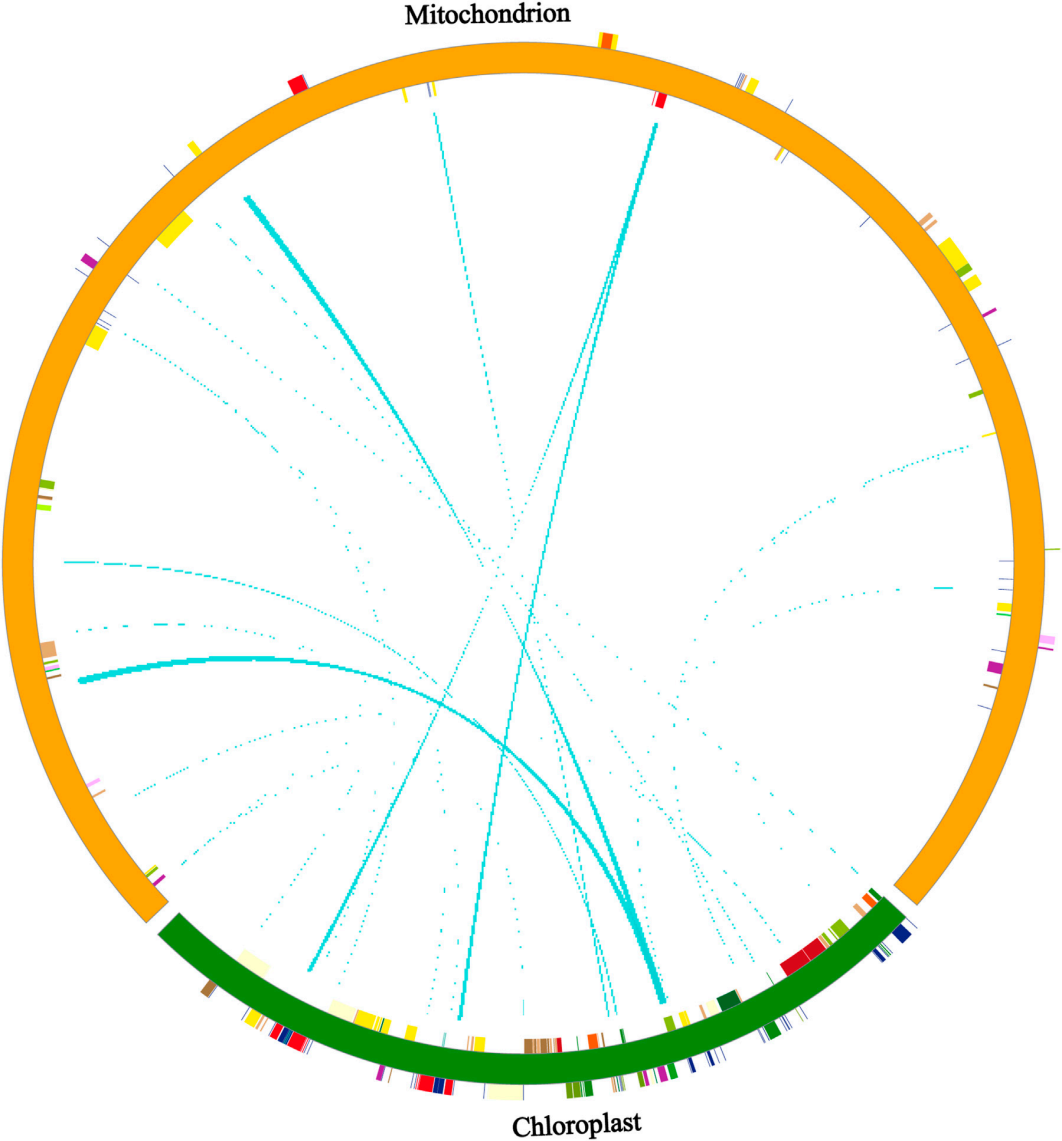
Distribution of homologous fragments of *P. tenella* chloroplast and mitochondrial genome sequences. Chloroplast sequences are chloroplast sequences, and the rest are mitochondrial sequences. Genes from the same complex are labeled with the same color, and the middle line connection indicates homologous sequences.

We analyzed DNA fragment migration between the chloroplast and mitochondrial genomes of *P. tenella*. A total of 45 pairs of homologous fragments were observed between the chloroplast and mitochondrial genomes of *P. tenella*, with 6,139-bp sequences in the chloroplast genome (3.89%) and 18,699-bp sequences in the mitochondrial genome (4.14%) (Figure 5; Supplementary Table S11). Furthermore, we identified protein-coding genes, tRNAs, and rRNAs transferred from the chloroplast genome to the mitochondrial genome. The results showed only seven tRNA genes were completely transferred, namely, *trnW-CCA*, *trnP-UGG*, *trnD-GUC*, *trnN-GUU*, *trnH-GUG*, *trnM-CAU*, and *trnI-CAU*. Conversely, protein-coding genes (*atpE*, *atpB*, *psbE*, *rpoB*, *psbC*, *atpA*, and *psbE*) and rRNA genes (*rrn16*) were all partially transferred (Supplementary Table S12).

### Phylogenetic analysis of mitochondrial genomes in higher plants

**FIGURE 6:**
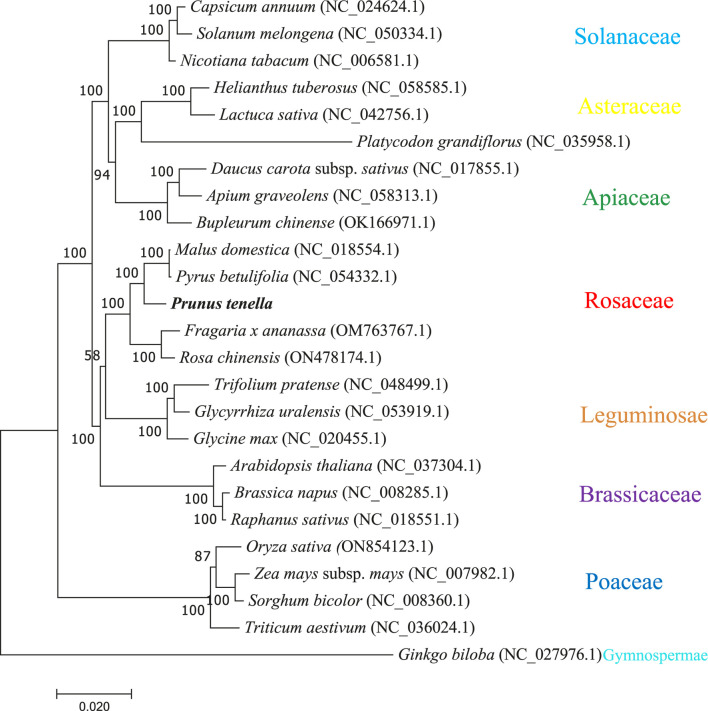
The phylogenetic tree constructed for *P. tenella* with 30 conserved genes in the mitochondrial genomes of 24 typical plants. Different colored dashed boxes represent plants of the same family.

To construct a phylogenetic tree, we selected 24 species and 30 protein-coding genes corresponding to each species. The phylogenetic tree was distributed according to seven families: Cruciferae, Leguminosae, Solanaceae, Gramineae, Rosaceae, Asteraceae, and Umbelliferae; species from the same family were highly clustered together, and one gymnosperm (*Ginkgo biloba*) was classified as an outgroup. In total, 21 of the 22 nodes generated by the phylogenetic tree showed Bootstrap support values > 70%, and 18 showed Bootstrap support values > 100% (Figure 6). The phylogeny strongly separated dicots from monocots (Poaceae) with a Bootstrap value of 100%. Among the dicotyledons, Rosaceae and Leguminosae are distantly related. Notably, *P. tenella* clustered conservatively with apples and pears belonging to Rosaceae, whereas strawberries and moonflowers clustered together, indicating that plant mitochondrial genomes differed among different genera in Rosaceae.

### Phylogenetic tree of mitochondrial genomes of *Prunus* species and *Ka*/*Ks* value analysis of protein-coding genes

**FIGURE 7:**
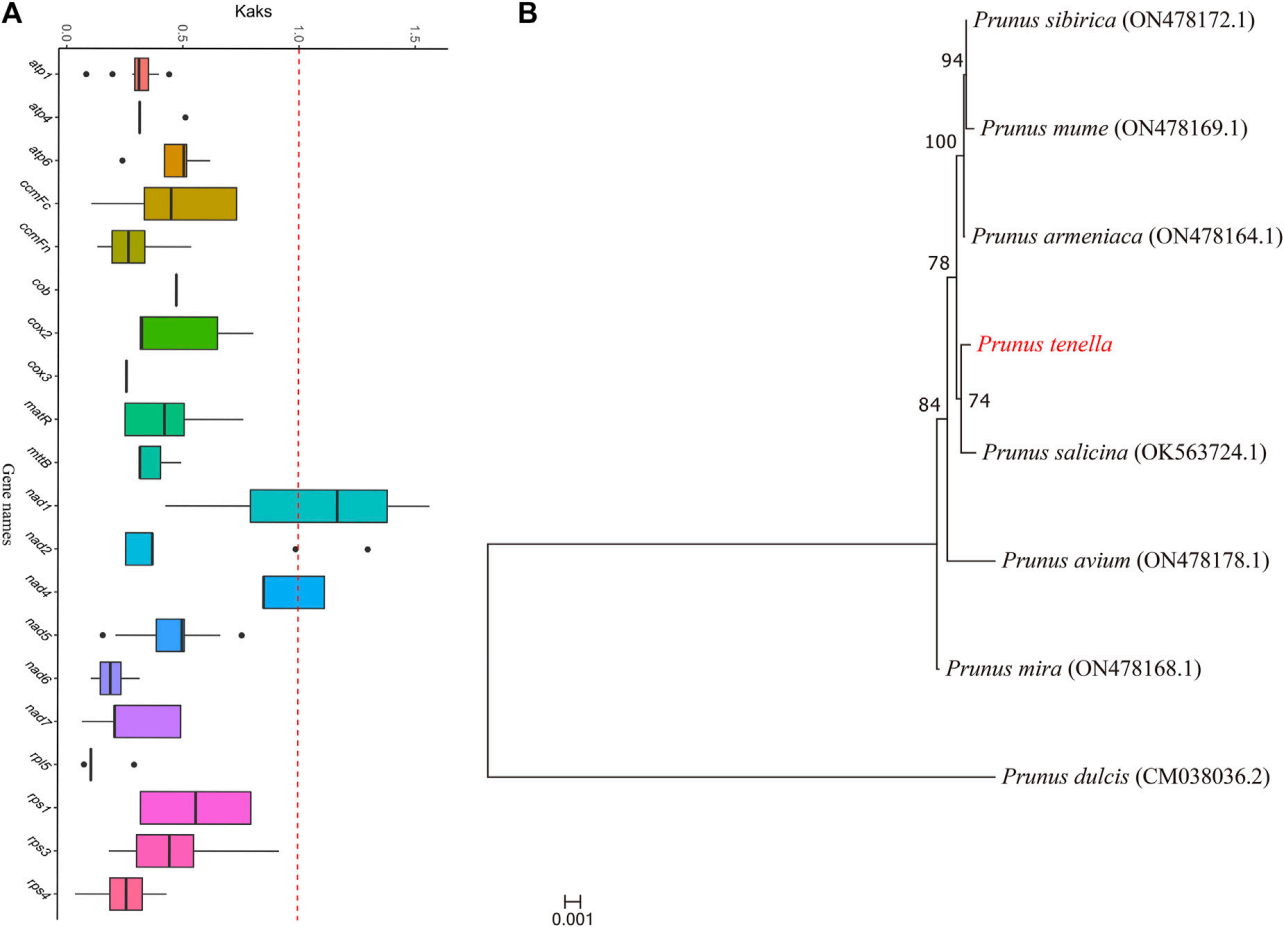
Phylogenetic tree and *Ka*/*Ks* values of protein-coding genes. **(A)** *Ka*/*Ks* values of 20 protein-coding genes obtained from eight *Prunus* species. Horizontal coordinates indicate gene names, whereas vertical coordinates indicate *Ka*/*Ks* values. The upper and lower endpoints of the vertical lines located above and below the rectangle in the box plot indicate the upper and lower edges of the data, respectively; the thick line inside the rectangle indicates the median; the upper and lower edges of the rectangle indicate the upper and lower quartiles, respectively; and the dots beyond the upper and lower edges of the data indicate outliers. Different colored dashed boxes represent plants of the same family. **(B)** Phylogenetic tree of the mitochondrial genomes of eight *Prunus* species.

We constructed a phylogenetic tree of eight *Prunus* species (Figure 7A). According to the clustering results, seven species, namely, *P. tenella*, *P*. *sibirica*, *P*. *mume*, *P*. *armeniaca*, *P*. *salicina*, *P*. *avium* and *P*. *mira*, clustered into one branch, whereas *P*. *dulcis* clustered into a separate branch. *P. tenella* is closely related to *P. salicina*. We calculated the *Ka*, *Ks*, and *Ka*/*Ks* values of 20 protein-coding genes in the mitochondrial genomes of eight *Prunus* species and plotted the box-line distribution (Figure 7B). The *Ka*/*Ks* values of 19 protein-coding genes were less than 1, indicating that these genes were highly conserved during the evolution of *Prunus* species and were subjected to purifying selection. In addition, *nad1* showed *Ka*/*Ks* values greater than 1, indicating that this gene was segregated during the evolution of the *Prunus* species and was subjected to positive selection.

## Discussion

Mitochondria are an energy source for plant and animal cells to carry out life activities; however, plant mitochondrial genomes are more complex than animal mitochondrial genomes (Kozik et al., 2019). Most plant mitochondrial genomes are over 1 Mb in size and contain diverse repetitive sequences. With advances in the organelle genome sequencing technology, numerous plant mitochondrial genomes are being sequenced and studied. Currently, mitochondrial genomes of several Rosaceae species have been studied, such as *P. salicina* (508,035 bp) (Fang et al., 2021), *P. avium* (444,576 bp) (Yan et al., 2019), *Rosa rugosa* (303,484 bp) (Park et al., 2020), and *Malus domestica* (396,947 bp) (Fang et al., 2021). These studies have shown that most Rosaceae members contain a highly conserved mitochondrial genome with a GC content of around 45%–46%. In the present study, we sequenced, assembled, and characterized the mitochondrial genome of the *P. tenella* resource “Yumin” species from the Xinjiang region, a national key grade II-protected wild plant in China. The sequencing and assembly results showed that the mitochondrial genome of *P. tenella* from Xinjiang was typically circular, with a length of 452,158 bp and a GC content of 45.55%. The mitochondrial genome of *P. tenella* was similar in length to that of *P. avium* and in the GC content to the Rosaceae mitochondrial genomes.

Most sequences in the plant mitochondrial genomes are non-coding, and most protein-coding genes contain ATG as the start codon (Ge et al., 2020). *P. tenella* mitochondrial genome was found to contain 36 protein-coding genes, of which 33 had ATG as the start codon, 2 (*nad1* and *rps4*) contained ACG and 1 (*rpl16*) contained GTG as the start codon. Protein-coding genes in the mitochondrial genomes of *Salix suchowensis*, *Phaseolus vulgaris*, and *Acer truncatum* Bunge also contained non-ATG-type start codons, a phenomenon known to be caused by RNA editing modifications (Sloan et al., 2018). Furthermore, we found multiple copies of *nad1*, *nad2*, *nad5*, and *rps4* in the *P. tenella* mitochondrial genome, similar to other Rosaceae mitochondrial genome studies (Bi et al., 2020b).

Repeated sequences, including tandem repeats, short repeats, and large repeats that are widely present in the mitochondrial genome, provide valuable information for developing markers for population and evolutionary analyses (Bi et al., 2016; Chung et al., 2017). The size of repeat sequences is closely related to the frequency of recombination. Although the frequency of recombination mediated by short repeats is lower than that mediated by long repeats, the ratio of their mediated heterodimers is almost equal (Fischer et al., 2022). In this study, we identified 142 SSRs, 26 pairs of tandem repeats, and 421 scattered repeats in the *P. tenella* mitochondrial genome. The presence of many repetitive sequences suggests that frequent intermolecular recombination events during the evolution of *P. tenella* possibly dynamically altered the structure and conformation of the mitochondrial genome (Chen et al., 2006). RNA editing processes in the chloroplast and mitochondrial genomes of higher plants occur post-transcriptionally, altering protein structures (Shearman et al., 2014). Previous studies have predicted 486 (31 protein-coding genes), 330 (33 protein-coding genes), and 491 (34 protein-coding genes) RNA editing sites in the mitochondrial genomes of *Phaseolus vulgaris*, *Salix suchowensis*, and *Oryza sativa*, respectively (Alverson et al., 2010). In the *P. tenella* mitochondrial genome, we predicted 507 RNA editing sites in 36 protein-coding genes. All the predicted sites in this study were located at the first and second codon positions and exhibited a C-to-U RNA editing pattern. These results are consistent with RNA editing sites and editing patterns reported in other plant mitochondrial genomes.

Advanced organelle genome sequencing has shown that most plants undergo unidirectional DNA fragment migration from the chloroplast genome to the mitochondrial genome. Only a few plants show a DNA transfer from the mitochondrial genome to the chloroplast genome (Notsu et al., 2002). In the present study, a total of 24,838-bp length sequences were found to be transferred from the *P. tenella* chloroplast genome (6,139 bp) to the mitochondrial (18,699 bp) genome. Seven protein-coding genes and one rRNA were partially transferred, whereas seven tRNA genes were transferred intact. Typically, complete tRNA gene transfer from the chloroplast to mitochondrial DNA occurs in plants such as soybean and *Acer truncatum* Bunge (Timmis et al., 2004).

We further phylogenetically analyzed the mitochondrial genomes of *P. tenella* by selecting 24 plants from the seven families. The high degree of conserved clustering of plants from the same family and the highly conserved clustering of *P. tenella* with Rosaceae plants such as apple and white pear indicated a low degree of mitochondrial genome segregation during the evolution of plants belonging to the same family. This result also shows that *P. tenella* belongs to the shrub category and is between herbaceous and arborous plants in Rosaceae family. Although both Rosaceae and Leguminosae belong to the order Rosales, a less conservative clustering of the species of the two families may be because of species differences, as Rosaceae contains apple and pear arbor plants, and Leguminosae comprises only herbaceous plants. Limited studies have investigated the clustering relationship of *P. tenella* in the genus *Prunus*. Molecular and morphological data for the clustering of *Prunus* species indicated that *P. persica* and *P. dulcis* belong to the *Amygdalus* ingroup, while *P. tenella* was identified as the outgroup (Bortiri et al., 2006). Based on *At103* gene sequence clustering, *P. tenella* and *Prunus divaricata* clusters were found to be highly conserved (Zhao et al., 2016). The phylogenetic results of the ITS sequence of ribosomal DNA showed that *P. tenella* and *P. persica* belong to two adjacent clades (Lee and Wen, 2001b). In addition, the phylogenetic results of the chloroplast genome sequences showed that *P. tenella* has a low genetic relationship with *P. persica* and *P. dulcis*. Our mitochondrial genome clustering results showed that *P. tenella* and *P. dulcis* belong to two branches, which is consistent with the clustering results of the above studies. However, mitochondrial genome clustering showed that *P. tenella* is highly conserved with six other *Prunus* species, which has not been reported in studies yet. This may be because fewer mitochondrial genomes of *Prunus* species were selected in the present study. Moreover, the *Ka*/*Ks* value of protein-coding genes showed that the mitochondrial genomes of *Prunus* are highly conserved. Future research should consider selecting more than 20 mitochondrial genomes of *Prunus* species to obtain more accurate results.

## Conclusion

In this study, we assembled and annotated the complete mitochondrial genome of *P. tenella*; the genome showed a typical circular structure, with a length of 452,158 bp. Sixty-three unique genes, including 36 protein-coding genes, 24 tRNA genes, and 3 rRNA genes, were identified in the genome. Additionally, this study examined codon usage, sequence duplication, RNA editing, and mitochondrial and chloroplast DNA fragment transfers in the *P. tenella* mitochondrial genome. The evolutionary status of *P. tenella* was verified through phylogenetic analysis by comparing the mitochondrial genomes of *P. tenella* and those of 24 other species. This study enriches the current understanding of the mitochondrial genome of *Prunus* species and provides a reference for subsequent studies on the evolution of *Prunus* species.

## Data Availability

The datasets presented in this study can be found in online repositories. The names of the repository/repositories and accession number(s) can be found in the article/Supplementary Material.

## Abbreviations

*atp1*, ATPase subunit 1 1; *atp4*, ATPase subunit 4; *atp6*, ATPase subunit 6; *ccmFc*, cytochrome c biogenesis FC; *ccmFn*, cytochrome c biogenesis FN; *cob*, apocytochrome b; *cox2*, cytochrome c oxidase subunit 2; *cox3*, cytochrome c oxidase subunit 3; *matR*, maturase; *mttB*, transport membrane protein; *nad1*, NADH dehydrogenase subunit 1; *nad2*, NADH dehydrogenase subunit 2; *nad4*, NADH dehydrogenase subunit 4; *nad5*, NADH dehydrogenase subunit 5; *nad6*, NADH dehydrogenase subunit 6; *nad7*, NADH dehydrogenase subunit 7; *rpl5*, ribosomal protein L5; *rps1*, ribosomal protein S1; *rps3*, ribosomal protein S3; *rps4*, ribosomal protein S4; *Ka*, nonsynonymous substitution rate; *Ks*, synonymous substitution rate; SSRs, simple sequence repeats.
