# Supplementary material for: Assembly and analysis of the complete mitochondrial genome of the Chinese wild dwarf almond (Prunus tenella)
Source: Front Genet. 2024 Jan 11;14:1329060. doi: 10.3389/fgene.2023.1329060 (PMC10811783; doi:10.3389/fgene.2023.1329060)
Supplement: Supplementary file 1 [file DataSheet1.ZIP › Supplementary figures.docx]

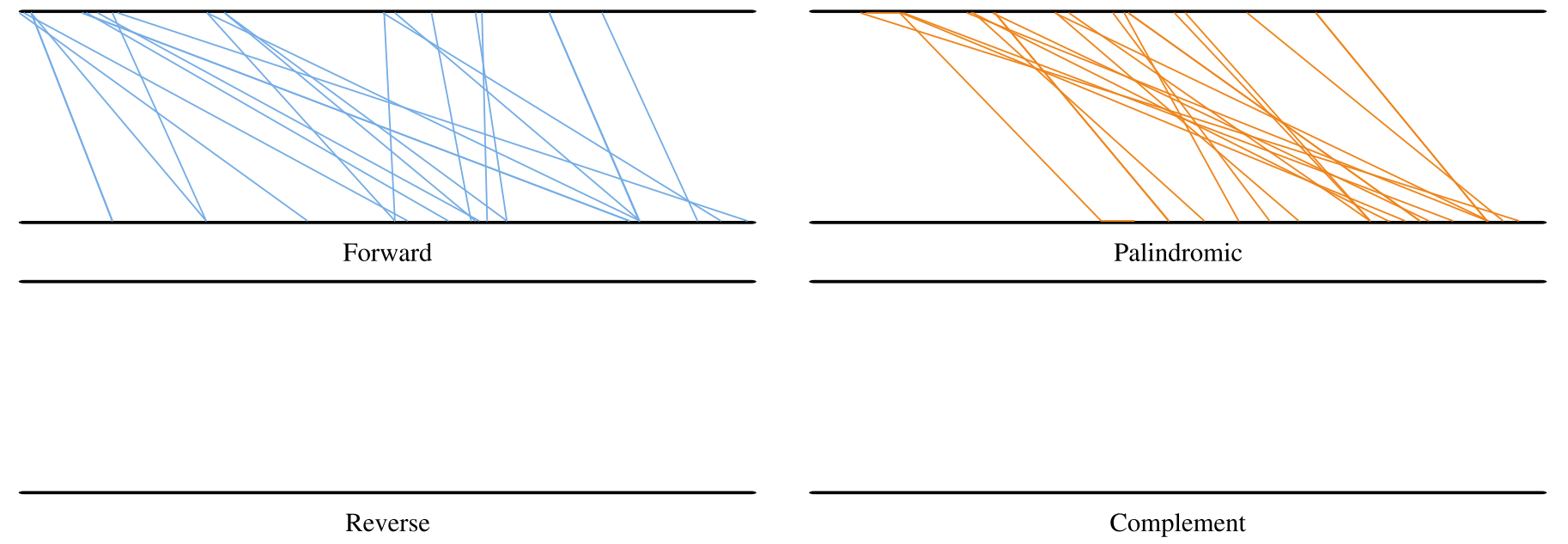


Figure S1: 100% repeat sequence fragment distribution location. The black parallel lines all indicate the mitochondrial genome of *Prunus tenella*. The blue line indicates the position of the Forward repeat sequence on the mitochondrial genome of *Prunus tenella*. The yellow line indicates the position of the Palindromic repeat sequence on the mitochondrial genome of *Prunus tenella*. No 100% repetitive sequences of Reverse and Complement types were found in the mitochondrial genome of *Prunus tenella*.


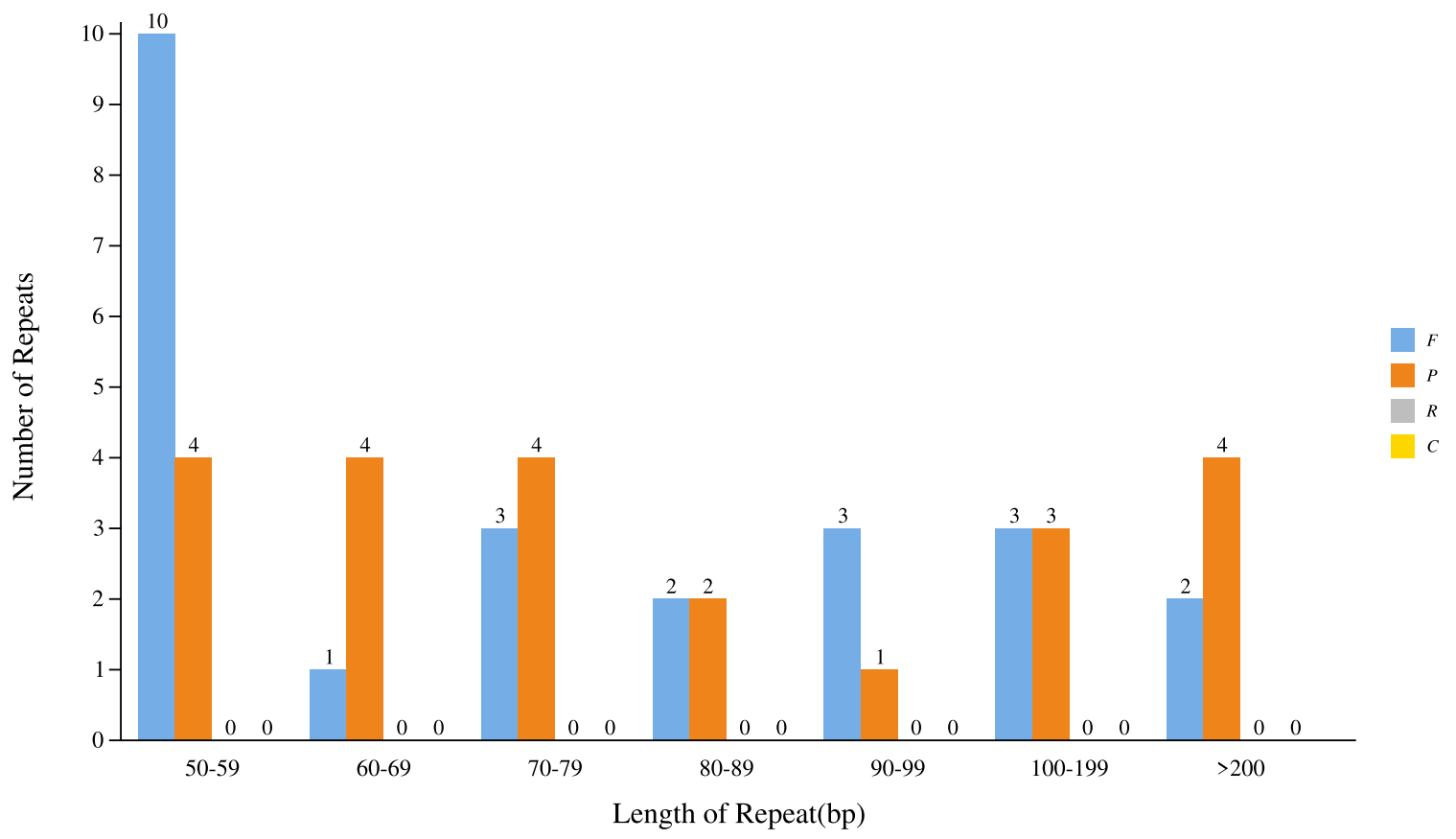


Figure S2: Statistical diagram of the distribution of different lengths of the four types of complete repeat sequences in forward, palindromic, reverse and complement in the mitochondrial genome of *Prunus tenella*. F=forward, P=palindromic, R=reverse, and C=complement. The abscissa represents the length of the complete repeat sequence. The vertical axis represents the number of complete repeat sequences.
